# The relationship between uploader identity and the quality and reliability of depression-related Chinese short videos on TikTok and Bilibili

**DOI:** 10.3389/fdgth.2026.1886505

**Published:** 2026-07-27

**Authors:** Xuechun Jiang, Furui Wang, Linglu Yu, Jinfeng Zhang

**Affiliations:** 1 Taicang Hospital of Nanjing University of Chinese Medicine (Taicang Hospital of Traditional Chinese Medicine), Taicang, China; 2 Nanjing University of Chinese Medicine, Nanjing City, Jiangsu, China; 3 Department of Neurology, Taicang Hospital of Nanjing University of Chinese Medicine (Taicang Hospital of Traditional Chinese Medicine), Taicang, China

**Keywords:** bilibili, depression, information quality, short videos, TikTok, upload identity

## Abstract

**Background:**

The depression has become a major challenge to global public health. Short video platforms like TikTok and Bilibili have become important channels for disseminating information about depression, but the quality and reliability have not been evaluated.

**Objective:**

This study aims to evaluate the quality and credibility of depression-related short videos on TikTok and Bilibili, focusing on the differences in the identity of the uploader and its impact on the reliability of the content.

**Methods:**

Using the keyword “depression” to collect 150 videos on TikTok and Bilibili. This research institute involves Chinese videos and Chinese uploaders. Divided The identity of uploaders into depressed patients, mental health educators, mental health practitioners and non-professional sharers. Use the Global Quality Score, modified DISCERN and JAMA benchmarking to evaluate.

**Results:**

Videos published by mental health practitioners have the highest JAMA scores and user participation indicators. The mDISCERN reliability score of non-professional sharing is the lowest. The content published by depressed patients is reliable, but the coverage and interaction are limited.

**Conclusion:**

The depression information constitutes a multi-party ecosystem, but the credibility depends on the identity of the uploader. Content from professional sources is still the most reliable. The platform should strengthen the authentication mechanism. Users should critically evaluate the source of content cautiously.

## 1 Introduction

The 11th edition of the International Classification of Diseases released by the World Health Organization classifies depression as a depressive disorder (1). As a psychological disease, depression is clinically characterized by persistent low mood or a significant decrease in interest and pleasure, accompanied by cognitive, behavioral or neurological symptoms, which can seriously damage the individual's socio-psychological function (2). There is evidence that the pathogenesis of depression involves the complex interaction between neurotransmitter disorders though, genetic susceptibility and environmental stressors, the current diagnostic practice still relies mainly on clinical interviews and standardized assessment scales (3). In the 2025 publication of World Health Organization *World mental health today: latest data*, it is noted that in 2021, depressive disorders alone were the second leading cause of global years lived with disability(YLDs), accounting for 6.2% of all YLDs (4). Severe depression is also one of the important factors in global incidence and mortality (5). In modern society, the accelerated pace of life and increased stress are closely related to the increase in the prevalence of depression (6).

Although public awareness of depression has increased recently, serious cognitive bias and social discrimination still exist (7). Simply attributing depression to “low mood” or “emotional vulnerability” ignores the complexity of depression as a biological psychosocial systemic disease. This misunderstanding leads to self-stigmatization and social prejudice (8), often delaying patients from seeking help and receiving appropriate treatment (9).

Increasing public awareness of mental health has now increased the demand for psychological counseling, especially among young people (10). They are more willing to seek professional help. Against this background, the short video platform has become an important channel for health communication and public education (11). Chinese mainstream platforms such as TikTok and Bilibili play a unique role in disseminating health information related to depression (12). These platforms are becoming increasingly important on the Internet. The video platform gathers rich resources covering causes, symptom identification, treatment plans and rehabilitation stories (13). Not only do these contents about depression have the potential to improve public mental health literacy, but they can also reduce social discrimination and build a mutual online community (14, 15).

However, there is still a lack of scientific and systematic research on the statistics of human identity characteristics and content generalization of producers of short videos related to depression at present. Existing studies rarely pay attention to how the identity of the uploader affects the credibility and dissemination effectiveness of information about depression (16). It may distort the understanding among the public and reinforce the misunderstanding of depression if the videos with wrong information about depression produced by non-professionals get the high dissemination in the complex information environment.

This study selects short videos related to depression on TikTok and Bilibili platforms, aiming to systematically analyze the identity background of content creators, and evaluate the scientific rigor and applicability of the videos through quality scoring. This study also tries to clarify how the identities of different uploaders affect the dissemination and reception of mental health information. It aims to provide supports for improving quality of mental health information on digital platforms, enhancing public mental health literacy, and promoting scientific conversations on clinical expertise.

## 2 Methods and materials

### 2.1 Ethical considerations of video selection

All the videos analyzed in this study come from the public content of TikTok and Bilibili, the two mainstream short video platforms in China. This research institute involves Chinese videos and Chinese uploaders. TikTok users mainly interact through likes, comments and shares, and the content of communication is often compressed into short and emotional expressions. And the user interaction of Bilibili is mainly carried out in the form of a bullet screen, which is a real-time scrolling comment system. Users can send comments while watching videos and superimpose them on the screen to create a feeling of “watching together”.

The research is strictly limited to public information and does not involve the personal privacy data of any uploader. Throughout the research process, the video content remained as it was and was not redistributed or used for commercial purposes. The content was only used as academic literature and evaluation materials. As a result, this research does not need to be approved by the institutional ethics committee and meets the standard ethical guidelines for research involving public digital content.

### 2.2 Time range of video selection

In order to accurately reflect the latest trends and transmission patterns related to depression more on social media platforms (17), the release time range of selected videos is limited to “the past year”, that is, from January 2025 to December 2025. Choosing this time range can reduce the potential deviation caused by the early release of the content, and also understand the recent attention to depression of public and the characteristics of information transmission.

### 2.3 Video search keyword settings

Using the core search term “抑郁症” to retrieve videos from TikTok and Bilibili platforms to ensure the consistency of the search criteria and the theme relevance of the analysis results. The keyword covers a wide range of content related to depression, depression knowledge and experience sharing of depressed patients. They can effectively reflect the mainstream video content related to depression disorders on the two platforms.

### 2.4 Characteristics of short video platforms

This study chooses TikTok and Bilibili as research platforms because their content forms and user interaction modes are different. TikTok is an algorithm-driven short video platform. It has highly fragmented, short and fast-paced, emphasizing real-time interactive content. In contrast, Bilibili mainly focuses on medium and long videos. The content structure is more systematic, usually including science popularization, experience sharing and knowledge interpretation.

The age coverage of TikTok is relatively wide, from teenagers to elderly people, covering the whole population. Bilibili users are highly concentrated on young and highly educated groups, and more inclined to content consumption and active learning. The user interaction mode is mainly based on collection and comments. These two platforms represent the two modes of “fast dissemination” and “deep content” in the mainstream short video category respectively, which are suitable for comparative analysis.

### 2.5 Video search process

**Figure 1:**
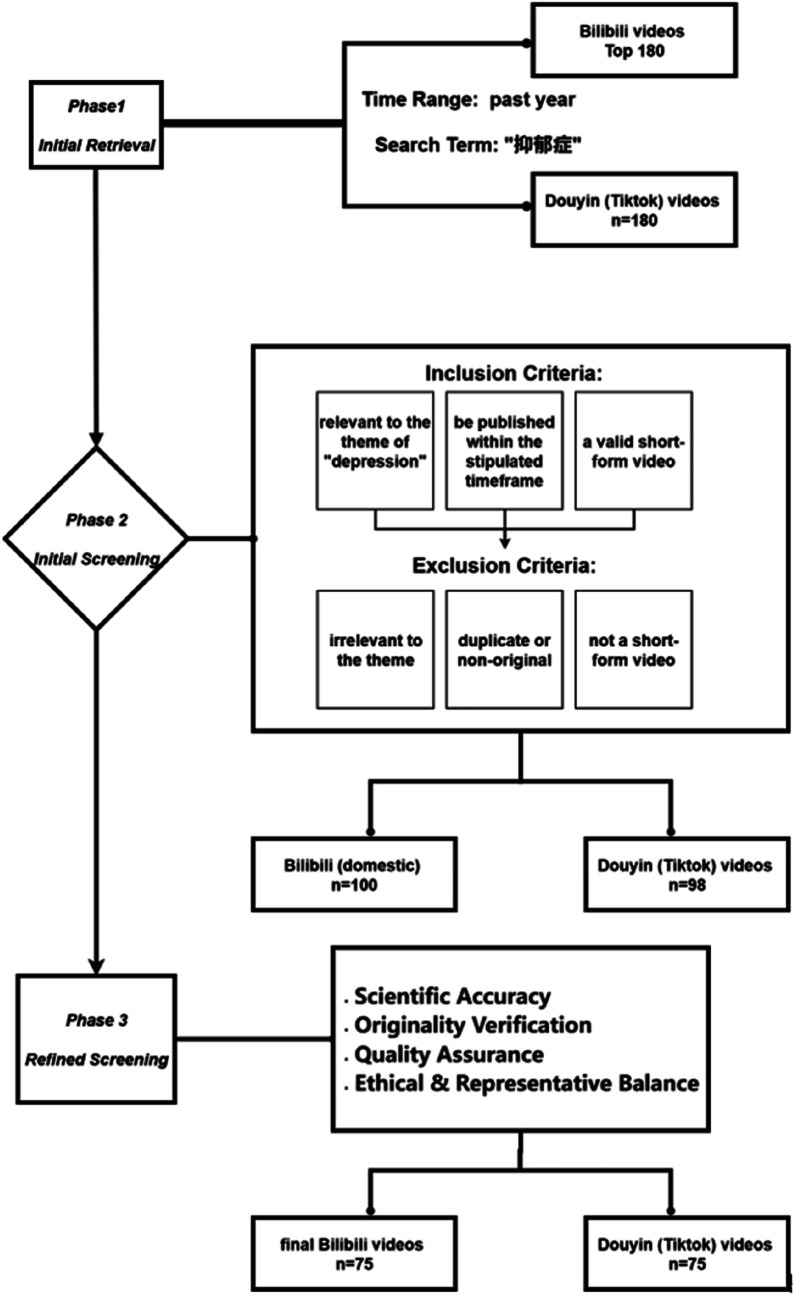
Search strategy for short videos on depression.

Searching the keyword “抑郁症” on TikTok and Bilibili for videos published from January to December 2025(past year) (Figure 1). To minimize potential bias introduced by platform-specific personalized recommendation algorithms, the study used newly registered “clean” accounts. Both platforms employed the default “comprehensive ranking” setting for video selection. The default ranking reflects the content that ordinary users are most likely to come into contact with in the context of nature use, which can be used as information to evaluate the actual impact of public cognition (18). However, due to the influence of the platform algorithm, the default ranking may tend to the highly interactive content recommended by the algorithm. Consistent with prior research, retrieving the top 180 videos ranked by this criterion using the search keyword on each platform provides a representative sample of the platform's mainstream content distribution (19), with a total of 360 videos as a preliminary screening pool.

### 2.6 Video filtering

A two-stage screening process was implemented. The core criterion for screening was that video content must be directly related to depression itself, the personal experiences or feelings of individuals with depression, or depression-related science popularization. The video screening of the two platforms is carried out by two researchers with medical background (FW and YL) according to the screening criteria to ensure the reliability of the screening results.

In the first stage, unrelated, repetitive, non-original videos, and those published outside the specified time range were excluded. Specifically, on TikTok, 54 unrelated videos and 28 non-original or repetitive videos were removed; on Bilibili, 62 unrelated videos and 18 non-original or repetitive videos were excluded.

In the second stage, the remaining videos underwent content review to further confirm thematic relevance and completeness. At this stage, 23 videos were excluded from TikTok and 25 videos from Bilibili.

In the end, we selected 75 videos as the final analysis samples. The 75 videos included in the analysis of the Bilibili platform come from 69 independent uploaders: 4 contributed 2 videos each, 1 contributed 3 videos, and the remaining 64 contributed 1 video each. The TikTok platform presents a similar distribution: out of 69 independent uploaders, 2 contributed 2 videos each, 2 contributed 3 videos each, and the remaining 65 contributed 1 video each. Therefore, the proportion of multiple videos of the same uploader is 14.67% in Bilibili and 13.33% in TikTok, which has little impact on statistical independence.

### 2.7 Choose the classification of video characteristics

For the 75 videos included in the analysis, we extracted the video lengths (in seconds) for recording. At the same time, the general information of the videos such as the number of likes, collections, comments and shares are recorded to quantify the coverage and user participation of the videos (20). The number of views on TikTok cannot be checked. In order to maintain cross-platform consistency, the playback volume is not included in the main analysis. Likes and shares can indirectly reflect video viewership, as engagement metrics correlate with algorithmic promotion and reach. Classify according to the main content of the selected video, including “expert psychoeducation”, “lived experience narratives”, “social advocacy”, “high-functioning depression”, “treatment modalities”, “metaphorical e representations” and “non- credible content”, and the content pattern analysis can be carried out in the future.

### 2.8 Uploader identity classification

In order to deal with overlapping identity characteristics, this study adopts hierarchical priority rules for mutually exclusive classification to ensure that each uploader is classified into only one category. The priority order is as follows: (1) Mental Health Practitioner: For professional qualifications of psychiatrists, psychotherapists and psychological counselors publicly certified by the platform, the official certification is more reliable than the self-declaration. (2) Individual with Depression: Depressed patients or convalescents who explicitly state in their videos or profile descriptions that they have been diagnosed with depression disorders or are currently in recovery. This category is based on the explanation of uploader. (3) Mental Health Education: Personal media accounts of psychology enthusiasts described by the platform as popular science bloggers. Their content explains depression from a psychological perspective, focusing on scientific popularization, symptom interpretation, and theoretical explanations of depression. (4) Non-Professional Sharing: individual media bloggers or lifestyle creators who do not have relevant professional knowledge. They share information based on independent learning and personal interests.

### 2.9 Video quality and reliability evaluation

This study uses internationally recognized Global Quality Score (GQS), Modified DISCERN (mDISCERN) and Journal of the American Medical Association (JAMA) benchmark criteria to score videos. GQS provides a comprehensive assessment of video quality about the overall structure, information integrity and educational value of the video. mDISCERN evaluates the reliability of information, the reference of authoritative sources, and the objectivity of content presentation from five specific dimensions. JAMA is used to evaluate the transparency and professionalism of online health information (21). The specific scoring details for GQS and mDISCERN are shown in Tables 1, 2 JAMA is represented in Table 3 All scores were completed independently by two researchers who have received standardized training and calibration tests (FW and YL). The evaluation process strictly follows the guiding principles of each tool to ensure the consistency and objectivity of the evaluation.

**Table 1:** The global quality score (GQS) quality criteria.

Item features	Points
Poor quality; poor flow of the videos; most information missing; not at all useful for patients	1
Generally poor quality; some information listed, but many important topics missing; of very limited use to patients	2
Moderate quality; suboptimal flow; some important adequately discussed, but other information poorly discussed; somewhat useful for patients	3
Good quality and generally good flow; most of the relevant information listed, but some topics not covered; useful for patients	4
Excellent quality and flow; very useful for patients	5

**Table 2:** The modified DISCERN (mDISCERN) quality criteria.

Reliability score
1. Is the video clear, concise, and understandable?
2. Are valid sources cited?
3. Is the content presented balanced and unbiased?
4. Are additional sources of content listed for patient reference?
5. Are areas of uncertainty mentioned?

**Table 3:** The journal of the American medical association (JAMA) benchmark criteria.

Score	Score component	
1 score	Authorship	Author and contributor credentials and their affiliations should be provided.
1 score	Attribution	Clearly lists all copyright information and states references and sources for content.
1 score	Currency	Initial date of posted content and subsequent updates to content should be provided.
1 score	Disclosure	Conflicts of interest, funding, sponsorship, advertising, support, and video ownership should be fully disclosed.

### 2.10 Statistical analysis

All statistical analysis is carried out using GraphPad Prism software (version 10.0). Since most of the variables of video information do not conform to the normal distribution, the continuous data is represented by the median and Interquartile Range (IQR) and written as M (Q1, Q3). For non-normal distribution variables, non-parametric tests are used between groups: Mann–Whitney U test is used for two groups, and Kruskal–Wallis test is used for three or more groups. Four uploader categories are dichotomized into a professional group and the other three categories combined a non-professional group, and compared them using the Mann–Whitney U test as a robustness check. This supplementary analysis helped test whether the main conclusions were sensitive to the classification. For a single hypothesis test, the significance level is set to *α* = 0.05. For multiple comparisons, Bonferroni is used to correct the control error rate. The significance level after correction is set to *α* = 0.05/3 ≈ 0.0167. Report the exact *P* value; *P* < 0.0167 is considered statistically significant.

## 3 Results

### 3.1 Identity distribution of video uploaders on TikTok and Bilibili

**Figure 2:**
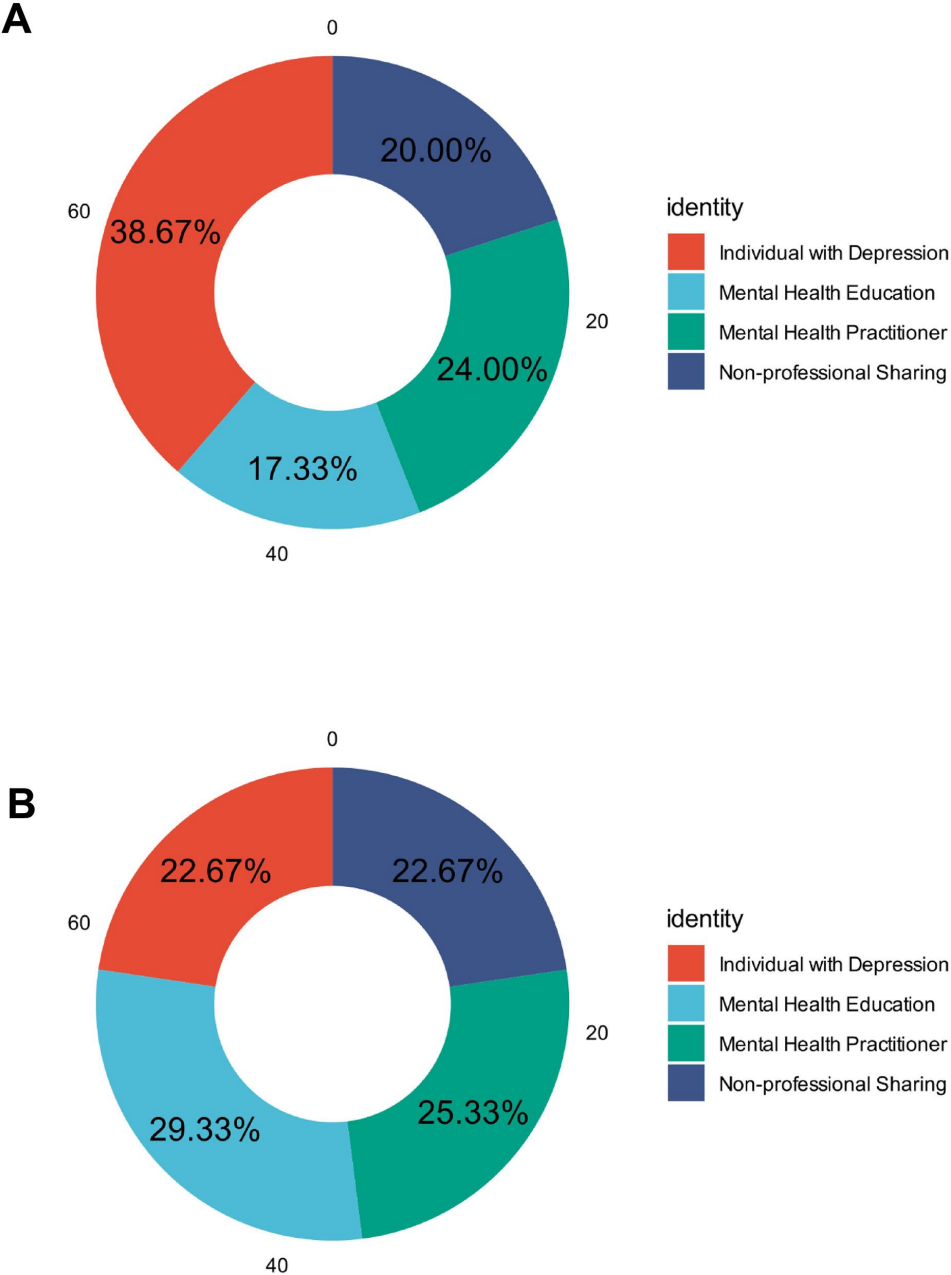
The percentage of different video uploader identities on TikTok and Bilibili. **(A)** Uploader identities on Bilibili. **(B)** Uploader identities on TikTok videos.

On the Bilibili platform Health Practitioner for 18 times (24.00%), Individual with Depression for 29 times (38.67%), Non-professional Sharing for 15 times (20.00%), Mental Health Education for 13 times (17.33%) (Figure 2A). Among the two video platforms, Bilibili is a platform that mainly focuses on medium and long videos and emphasizes effective interaction, showing a relatively obvious tendency in the content of videos about depression. The perspective of the patients is relatively prominent (38.67%), which reflects the high acceptance and emotional resonance of the user group to the “first-person narrative”, and also reflects the emphasis on the authenticity of personal experience in the topic of mental health on the platform. The proportion of professionals remains stable (24.00%), indicating that the voice of experts continues to participate in the public discussion on the platform.

On the TikTok platform, the identity distribution of uploaders of depression-related videos Mental Health Practitioner for 19 cases (25.33%), Individual with Depression for 17 cases (22.67%), Non-professional Sharing for 17 cases (22.67%), Mental Health Education for 22 cases (29.33%) (Figure 2B). As a rapid and interactive short video platform, TikTok is more inclined to promote emotional resonance and real-time dissemination through videos pushed by big data. Popular science content about depression accounts for the largest proportion on TikTok, reflecting the great demand of users for structured and health knowledge which is easy to understand. At the same time, mental health practitioners are also actively using short videos to carry out public mental health education and introduce the treatment of depression. This can reduce social discrimination against mental illness.

### 3.2 Distribution of uploader identity of all videos on TikTok and Bilibili

**Figure 3:**
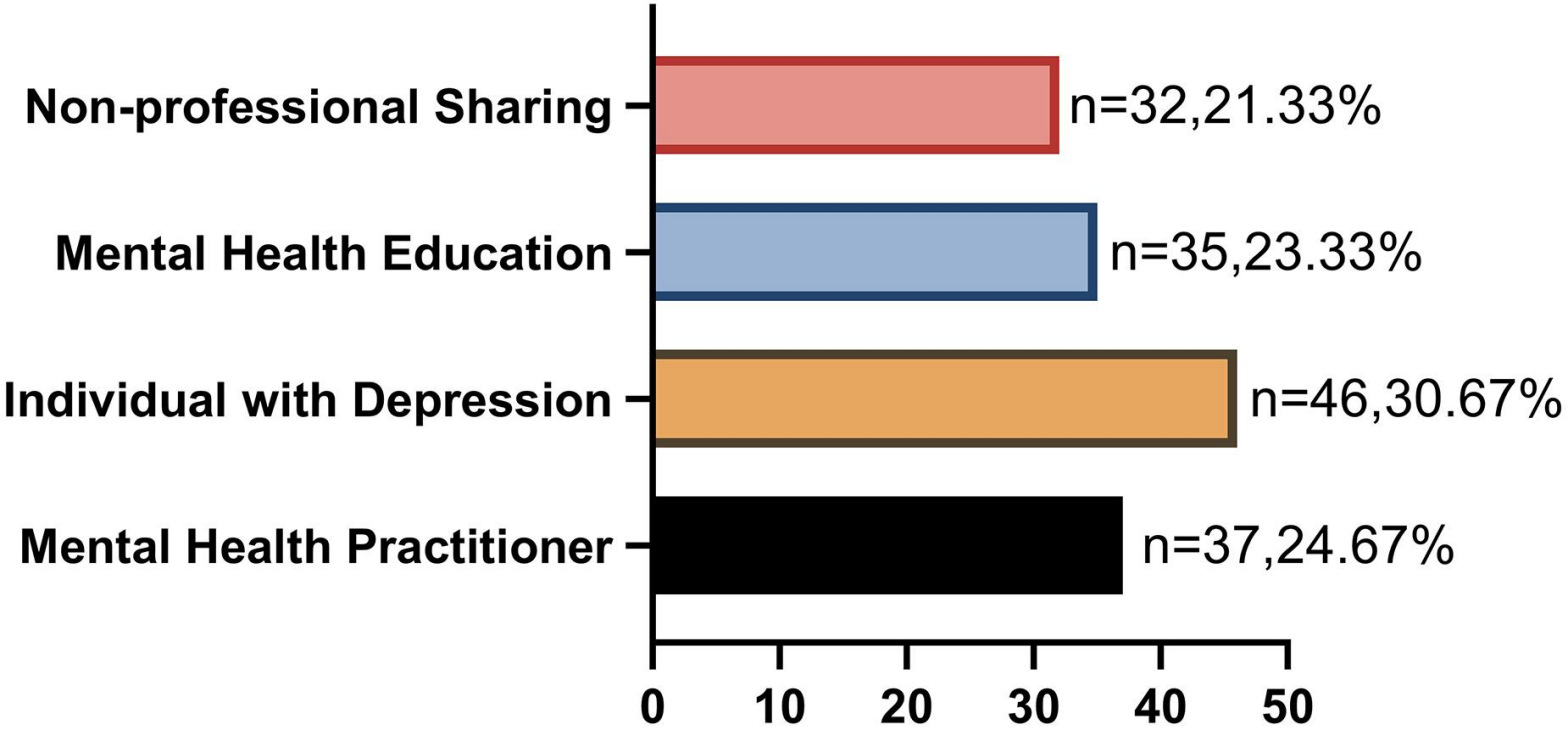
Different uploader identities by count on TikTok and Bilibili.

The 150 videos about depression collected from TikTok and Bilibili divide the identities of all video uploaders on the two platforms into four categories for integrated analysis (Figure 3). Detailed content classification of the videos is provided in Supplementary Table S1A and Supplementary Figure S1A. The difference in the number of different types of video content will be influenced by the focus of knowledge sharing, emotional expression and social advocacy in terms of content production motivation. Secondly, creators will produce this according to which content is more attractive to the audience. The threshold of video creation will also affect the number of different types of videos, professional knowledge, expression ability and production cost.

Among them, the number of videos uploaded by Individual with Depression is the largest, with a total of 46, accounting for 30.7% of the total sample. In some videos, Individual with Depression record the treatment experience of the disease or the manifestations of the onset of depression, and some rehabilitation personnel recall their treatment process. Followed by Mental Health Practitioners (*n* = 37, 24.7%) and Mental Health Education (*n* = 35, 23.3%), Non-professional Sharing uploaded the least number of videos, with a total of 32, accounting for 21.3% of the data set. This distribution reflects the diverse participation mode of content creation on two platforms, both from the perspective of personal experiences and professionals. In the video platform, diverse interpretation and dissemination of related knowledge can be formed, so that relevant people can publish videos to record their lives and share knowledge. It can reduce public ignorance of depression, and also reduce the stigma of mental illness.

### 3.3 Basic video information and video quality scores of TikTok and Bilibili

Recording the general information and quality score of the videos (Table 4). Regarding the video duration, the median of Bilibili is 212.00 s (IQR: 143.00–601.00), which is much longer than the seconds on TikTok (M = 68.00, IQR: 33.00–148.00). This difference shows that videos related to depression on the Bilibili platform tend to provide more detailed, complete and rich content, and can introduce depression in multiple dimensions; while videos on the TikTok platform are more refined in content, conveying core views or emotional experiences in a relatively short time.

**Table 4:** General information, quality, and reliability scores of videos on TikTok and Bilibili.

Variables	Bilibili (*n* = 75)	TikTok (*n* = 75)	*P*
General information			
Video length(s), M (Q1, Q3)	212.00 (143.00, 601.00)	68.00 (33.00, 148.00)	*P* *<* *0.0001*
Likes, M (Q1, Q3)	2,516.00 (794.00, 8,554.00)	2,50,270.00 (56,748.00, 4,11,785.00)	*P* *<* *0.0001*
Collections, M (Q1, Q3)	1,587.00 (564.00, 3,949.00)	9,201.00 (4,118.00, 22,414.00)	*P* *<* *0.0001*
Comments, M (Q1, Q3)	71.00 (21.00, 207.00)	25,103.00 (7,842.00, 59,797.00)	*P* *<* *0.0001*
Shares, M (Q1, Q3)	156.00 (47.00, 607.00)	40,971.00 (8,459.00, 1,20,443.00)	*P* *<* *0.0001*
Video quality			
GQS score, M (Q1, Q3)	3.00 (3.00, 4.00)	3.00 (3.00, 3.00)	*P* *=* *0.9586*
mDISCERN score, M (Q1, Q3)	3.00 (3.00, 3.00)	2.00 (2.00, 3.00)	*P* *<* *0.0001*
JAMA score, M (Q1, Q3)	2.00 (2.00, 2.00)	2.00 (2.00, 2.00)	*P* *=* *0.0553*

**Figure 4:**
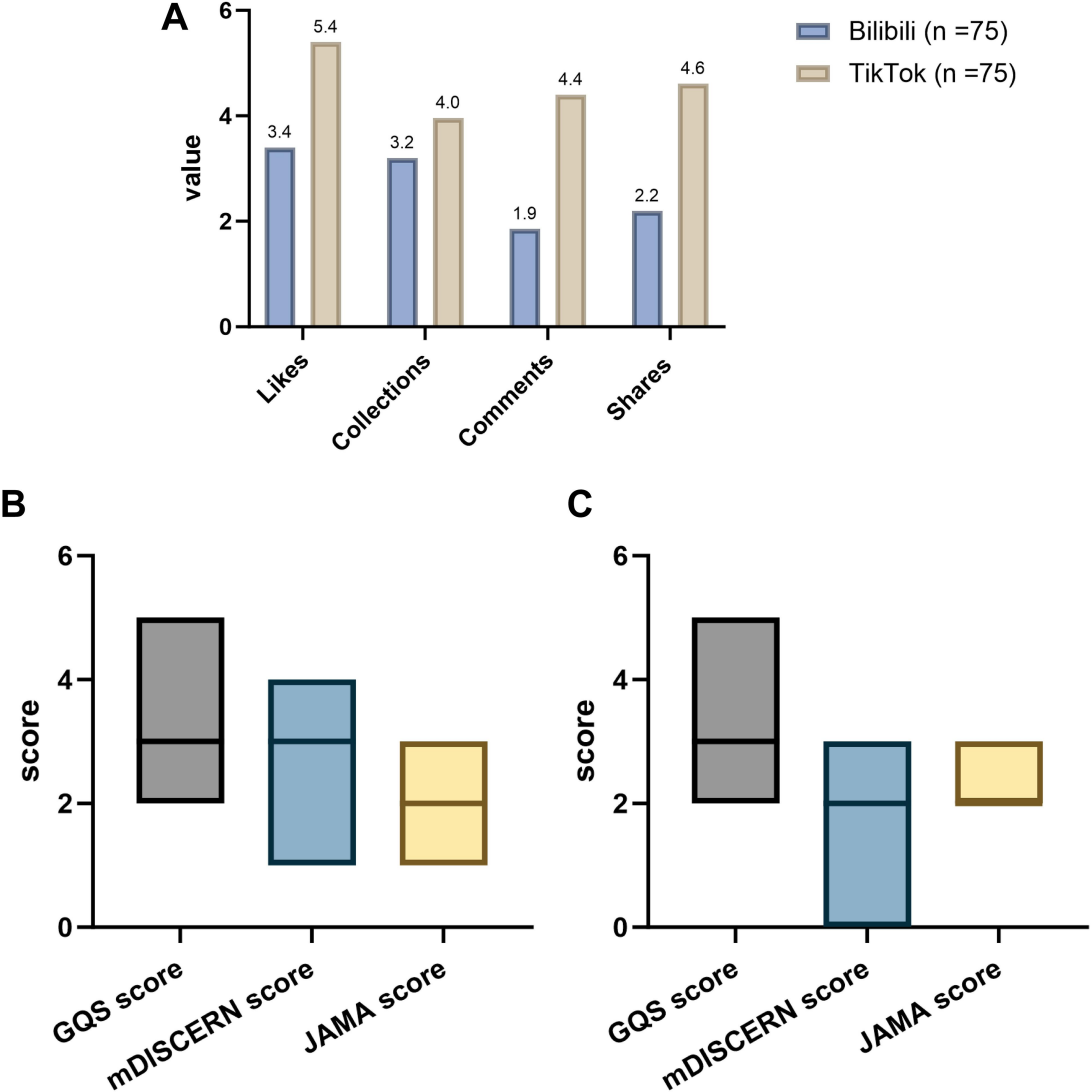
General video information data on Bilibili and TikTok. **(A)** General information comparison of videos on TikTok and Bilibili, transformed according to log(M + 1). **(B)** Quality scores (GQS, mDISCERN, JAMA) for Bilibili. **(C)** Quality (GQS, mDISCERN, JAMA) scores for TikTok videos.

In view of the significant numerical differences in the variables of each participation index, in order to better visualize and analyze, the median of the interactive indicators was converted to log10 (M + 1) to characterize the participation level on each platform. (Figure 4) In terms of user interaction, the median number of likes on TikTok reached 250,270.00 (IQR: 56,748.00–411,785.00), which is about Bilibili (2,516.00; IQR: 794.00–8,554.00) ten times. Other interactive indicators also show a similar trend: median number of collections (TikTok: 9,201.00; Bilibili: 1,587.00), comments (TikTok: 25,103.00; Bilibili: 71.00) and shares (TikTok: 40,971.00; Bilibili: 156.00). These findings show that algorithm recommendation system and user participation mechanism of TikTok can effectively promote the rapid and wide range online dissemination of content.

Regarding video quality, the evaluation results of different judging criteria are different (Figures 4B,C) here is no significant difference in GQS scores of the two platforms (*P* = 0.9586), The median value of both Bilibili and TikTok is 3.00. The video quality of both platforms can meet the standard of medium and above. However, the mDISCERN score shows that score of Bilibili (M: 3.00; IQR: 3.00–3.00) is significantly better than TikTok (M: 2.00; IQR: 2.00–3.00; *P* < 0.0001), indicating that the videos on Bilibili show higher reliability in terms of information source and content rigor. In comparison, there is no significant difference in the JAMA benchmark score between groups (*P* = 0.0553), the median of Bilibili is 2.00 (IQR: 2.00–2.00), and that of TikTok is 2.00 (IQR: 2.00– 2.00).

### 3.4 Quality scores of different uploader videos on two platforms

**Figure 5:**
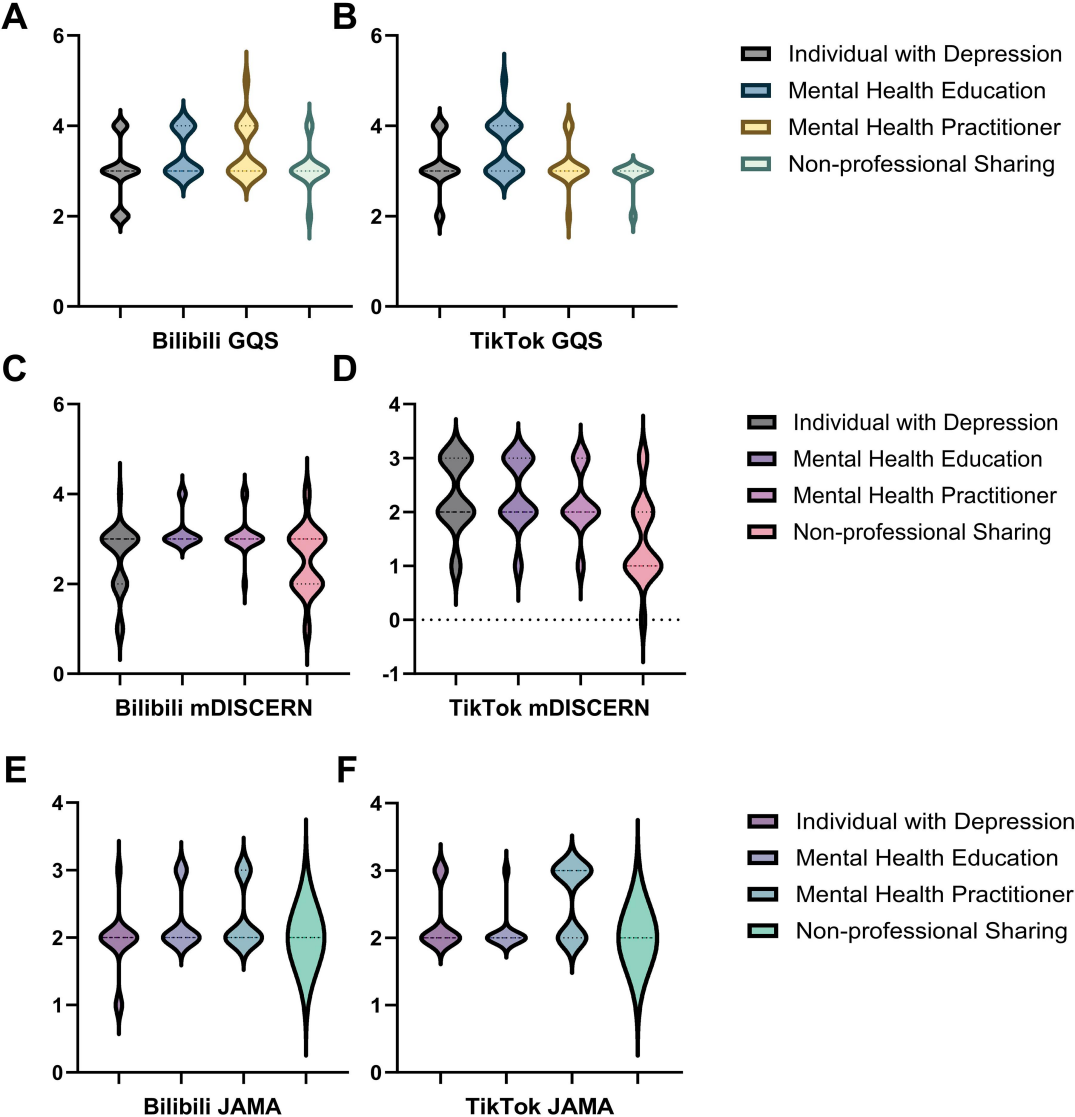
Quality scores (GQS, mDISCERN, JAMA) of videos from different uploaders on TikTok and Bilibili across platforms. **(A)** GQS scores on Bilibili. **(B)** GQS scores on TikTok. **(C)** mDISCERN scores on Bilibili. **(D)** mDISCERN scores on TikTok. **(E)** JAMA scores on Bilibili. **(F)** JAMA scores on TikTok.

The Mental Health Practitioners show clear advantages in video quality (GQS) (Figure 5A), its third quartile (Q3 = 4) is significantly higher than that of depressed patients (*P* = 0.006). This shows that professional training and resource investment help to improve the audio-visual structure and presentation effect of the video. Quality analysis of videos with the identity of different uploaders on TikTok found that mental health educators performed outstandingly in video production quality, the GQS scores (M = 3.5, *P* = 0.021) (Figure 5B), and have strong audio-visual expression in the identity of TikTok educators. Ability and rhythm control can be well matched with the communication logic of short videos.

Although no significant overall difference was observed in information reliability (the mDISCERN scores represent) on Bilibili (*P* = 0.056) (Figure 5C), but the first quartile (Q1 = 3) of mental health practitioners is higher than that of depressed patients (Q1 = 2), which shows that professionally produced content maintains a more stable baseline in terms of evidence citation and information objectivity. However, in terms of mDISCERN scores on TikTok (Figure 5D), the scores of all creator groups are generally lower (M = 2), and the difference between groups is significant (*P* = 0.034). This shows that sometimes the excessive pursuit of communication efficiency will lack the rigor of information and the support of evidence.

The most distinctive indicator is the JAMA scores on Bilibili (Figure 5E), mental health practitioners are significantly superior to other groups in this indicator (*P* < 0.001, Q3 = 3), indicating that their content more strictly follows academic communication ethics, including author identity transparency, source labeling and conflict of interest disclosure. In terms of academic rigor on TikTok (Figure 5F), only mental health practitioners maintained a relatively high level (M = 3), while other groups had lower scores. This further shows that the normative requirements on such platforms have not reached a general consensus and rely more on the professional self-discipline and habitual practices of creators.

### 3.5 Video overview and video quality rating of different uploader identities

The video quality scores of different uploaders on Bilibili and TikTok platforms are in Table 5. The results showed that the differences between Mental Health Practitioner and other three types of uploaders showed obvious dimensional dependence. In terms of GQS, Mental Health Practitioner are significantly superior to Individual with Depression (*P*_1_ = 0.0005) and Non-professional Sharing (*P_3_* = 0.0101), but there is no statistical significance between them and Mental Health Education (*P*_2_ = 0.3826), indicating that educators who have been engaged in popular science creation for a long time can also reach a level comparable to that of professionals in terms of production quality. In terms of mDISCERN, the results are similar. Mental Health Practitioner are significantly better than Individual with Depression (*P*_1_ = 0.0025) and Non-professional Sharing (*P*_3_ = 0.0141), while there is almost no difference between them and Mental Health Education (*P*_2_ = 0.8900), and this *P* value is high. In terms of JAMA, Mental Health Practitioner are only significantly superior to Individual with Depression (*P*_1_ = 0.0407), and the difference between Mental Health Education (*P*_2_ = 0.2936) and Non-professional Sharing (*P*_3_ = 0.0952) is not statistically significant.

**Table 5:** Quality scores (GQS, mDISCERN, JAMA) of videos by different uploader identities on Bilibili and TikTok.

Variables	Mental health practitioner (*n* = 18)	Individual with depression (*n* = 29)	Mental health education (*n* = 13)	Non-professional sharing (*n* = 15)	*P*
Video quality (Bilibili)					
GQS score, M (Q1, Q3)	3.00 (3.00, 4.00)	3.00 (2.50, 3.00)	3.00 (3.00, 4.00)	3.00 (3.00, 3.00)	*P_1_* *=* *0.0005*
					*P_2_* *=* *0.3826*
					*P_3_* *=* *0.0101*
mDISCERN score, M (Q1, Q3)	3.00 (3.00, 3.00)	3.00 (2.00, 3.00)	3.00 (3.00, 3.00)	3.00 (2.00, 3.00)	*P_1_* *=* *0.0025*
					*P_2_* *=* *0.8900*
					*P_3_* *=* *0.0141*
JAMA score, M (Q1, Q3)	2.00 (2.00, 3.00)	2.00 (2.00, 2.00)	2.00 (2.00, 2.50)	2.00 (2.00, 2.00)	*P_1_* *=* *0.0407*
					*P_2_* *=* *0.2936*
					*P_3_* *=* *0.0952*

**Variables**	**Mental Health Practitioner (*n* = 19)**	**Individual with Depression (*n* = 17)**	**Mental Health Education (*n* = 22)**	**Non-professional Sharing (*n* = 17)**	***P***
Video quality (TikTok)					
GQS score, M (Q1, Q3)	3.00 (3.00, 3.00)	3.00 (3.00, 3.50)	3.50 (3.00, 4.00)	3.00 (3.00, 3.00)	*P_1_* *=* *0.0110*
					*P_2_* *=* *0.8920*
					*P_3_* *=* *0.0002*
mDISCERN score, M (Q1, Q3)	2.00 (2.00, 3.00)	2.00 (2.00, 3.00)	2.00 (2.00, 3.00)	1.00 (1.00, 2.00)	*P_1_* *=* *0.3683*
					*P_2_* *=* *0.1692*
					*P_3_* *=* *0.0103*
JAMA score, M (Q1, Q3)	3.00 (2.00, 3.00)	2.00 (2.00, 2.50)	2.00 (2.00, 2.00)	2.00 (2.00, 2.00)	*P_1_* *=* *0.1652*
					*P_2_* *=* *0.1993*
					*P_3_* *=* *0.0473*

On the TikTok platform shows that the difference between Mental Health Practitioner and the other three types of uploaders shows a different pattern from that on the Bilibili platform. First of all, in terms of video production quality (GQS), the results display that the difference between Mental Health Practitioner and Non-professional Sharing is the most significant (*P*_3_ = 0.0002), and there is also a significant difference between Individual with Depression (*P*_1_ = 0.0110). In contrast, the difference between Mental Health Practitioner and Mental Health Education did not reach statistical significance (*P*_2_ = 0.8920). This result shows that on the TikTok platform, the video production quality of Mental Health Practitioner is significantly better than that of depressed patients and Non-professional Sharing. Secondly, there is a significant difference between Mental Health Practitioner and Non-professional Sharing alone (*P*_3_ = 0.0103) in the mDISCERN, while the difference between Mental Health Practitioner and Individual with Depression (*P*_1_ = 0.3683) and Mental Health Practitioner and Mental Health Education (*P*_2_ = 0.1692) is not statistically significant. Thirdly, in terms of JAMA, the differences between mental health practitioners and the other three groups lack statistical significance.

## 4 Discussion

### 4.1 The Influence of uploader identity on the dissemination and reception of mental health information

The present study institute involves Chinese videos and Chinese uploaders and demonstrates that significant differences across uploader types indicate that who delivers the information is as important as the content itself. Judging from the professional background of content producers, mental health practitioners usually have systematic professional knowledge and clinical experience, and their identity gives their video content higher authority and credibility (22). Users are more inclined to trust and disseminate such content uploaded by professional verifiers, which significantly improves the collection and sharing rate of videos (23). Let professional practitioners undertake the dissemination of systematic knowledge popularization and evidence-based treatment information; mental health educators carry out depression science popularization creation (24).

However, emphasizing the importance of professional content does not mean denying the value of non-professional content and personal experience sharing. Although non-professional sharers lack officially certified qualifications, their content is usually based on the accumulation of personal knowledge and has a strong narrative appeal, so that they can obtain a wider range of interactive participation (25). This aligns with existing findings in Social Media Analytics suggesting that user-generated content tends to stimulate higher levels of participatory engagement, even if its dissemination reach is comparatively limited (26). At the same time, the content shared by depressed patients is mainly focused on expression and emotional catharsis. Although this kind of content has the advantage of authenticity, it is often too personal and emotional, so it is difficult to attract wide participation (27). A healthy mental health transmission ecology should be a hierarchical synergy. Professional practitioners are most suitable for disseminating systematic knowledge and evidence-based treatment information, while non-professional sharers attract a wider audience through narratives close to life and lower the psychological threshold for the public to contact the topic of depression. It is recommended that the platform can guide users to pay priority attention to content from professional certification sources and expand the coverage and social influence of mental health information. At the same time, personal narrative content is allowed to provide space for emotional support and peer connection. This balance strategy can maintain scientific rigor. In addition, the findings of this study can provide data support for content review, quality evaluation and algorithm optimization of short video platforms. Platforms can consider introducing a quality scoring mechanism in mental health content, reducing the weight of recommendations, and promoting the formation of a healthier and more orderly digital information ecology.

### 4.2 Differences in video quality scores between the two platforms

There is no statistically significant difference in the overall perceived quality of the two platforms (GQS median: Bilibili 3.50 vs. TikTok 3.00, *P* = 0.5963). This shows that from the perspective of ordinary audiences watching videos, the videos on both platforms are generally in a good position in terms of production quality, narrative fluency and ease of understanding content.

However, in the evaluation of information reliability, the score of Bilibili (median 3.00, quartile spacing: 2.50–3.50) is significantly higher than TikTok (median 2.00, quartile spacing: 1.50–2.50). A higher mDISCERN score usually indicates that videos pay more attention to citing verifiable sources (28). The main root cause of the difference is the different identity composition of the uploaders of the two platforms. On Bilibili, mental health practitioners and mental health educators account for a larger proportion. They will pay more attention to the accuracy of information when producing content. Mental health professional practitioners who have received long-term academic training will systematically pursue the transparency of information sources, so they have a significant advantage in terms of content credibility and authority (29). In contrast, the proportion of depressed patients and non-professional sharers on TikTok is higher. In the TikTok system, its platform characteristics sometimes affect the quality structure of videos on the topic of mental health (30). The algorithm mechanism of TikTok is more inclined to recommend content with high interaction rate and strong emotion, which will allow creators to adopt a more emotional expression, further widening the gap between the two platforms in information rigor. Therefore, the mental health content on TikTok shows the characteristics of form over content (31).

According to the content authority of the JAMA benchmark evaluation, the median score of TikTok (2.00) is slightly higher than Bilibili (1.50), but the difference is not statistically significant. The scores show that a considerable part of short videos about mental health fail to fully disclose their background, lack professionalism, and reduce authority (32).

These findings show that in the current video environment, the dissemination effect of video content on the two platforms is not positively related to whether it meets the professionally recognized reliability standards (33).

The persuasiveness of information depends on the professionalism and reliability of the source. This study provides empirical support for the content advantages of mental health practitioners. On platforms driven by algorithm, there is a complex nonlinear relationship between information quality and dissemination effect. While pursuing the breadth of communication in the practice of health communication, how to maintain the quality of information is an issue that needs to be carefully weighed.

### 4.3 Differences in video interaction between the two platforms

The data of this study reveals the differences in the interaction of depression-related health information on two video platforms, TikTok and Bilibili. On TikTok, short videos are characterized by extremely short duration, high user participation, and the median number of likes, comments and shares can reach hundreds of thousands. This indirectly determines the communication logic of TikTok, which is to quickly trigger emotional resonance and promote social interaction as the core (34). Consequently, TikTok has constructed a communication mode centered on instant emotional exchange (35).

In contrast, the median number of video interactions on Bilibili is much lower than that of TikTok, but the rate of collections and likes is significantly higher. This makes Bilibili look more like an informal learning community (36).

### 4.4 Future research direction

Future research can explore the matching between different uploader identities and video content categories (37). For example, whether expert uploaders mainly produce authoritative interpretation content for expert knowledge sharing, and whether ordinary users are more content such as life experience descriptions or treatment patterns, further study how the uploader's identity affects content distribution. And study how the matching of uploaders and video content on different platforms affects audience participation and knowledge acquisition, which can provide insights for effective mental health communication videos and better formulate targeted and empirical-based depression-related content dissemination strategies. In addition, when selecting videos, the data of the number of views should be uniformly obtained on the platform, and the number of plays should be included in the research content.

### 4.5 Limitations of this study

The relatively short research cycle cannot reflect the dynamic changes of the platform algorithm and the content ecosystem. The relevant data statistics are not comprehensive enough. There are two videos contributed by the same uploader in the sample of this study, which has a small potential impact on statistical independence. Future studies still recommend different sampling strategies to completely eliminate this potential bias. Content classification relies on manual judgment, which will introduce subjective bias. In addition, this study is limited to the Chinese platform, which limits the cross-cultural applicability of the research results.

## 5 Conclusion

This study collected and evaluated the information quality of 150 videos about depression from TikTok and Bilibili. The research results show that the content of professional sources shows excellent reliability and standards. Based on the evidence of these platforms, a multi-participant and functionally complementary communication environment has been formed around depression-related topics. Depressed patients share real personal narratives, while mental health practitioners provide scientific, rigorous and systematic knowledge. Users should give priority to the content of authoritative institutions as preliminary reference materials, not the basis for diagnosis or treatment.

Collaborative interaction between different uploader identities helps to raise public awareness of depression. However, this diversified structure also leads to the stratification of information credibility. The content of professional sources has more authoritative clinical guidance value and shows higher user participation. In contrast, although the content from non-professional sharers resonates emotionally, it often lacks evidence to support it and gets a lower reliability score.

These results show that a hierarchical and responsible collaborative public discourse environment must be built in the information age. Professionals undertake the core functions of knowledge verification and authoritative guidance. Public communication can take advantage of the coverage of short video platforms to convey evidence-based information in an easy-to-understand but rigorous way. The platform needs to clarify the identity of the uploader to help ordinary users establish information identification ability and identify reliable content. Through the quality improvement of the professional supply side and the construction of information literacy on the public demand side, a public discussion space for depression that can maintain vitality and credibility is formed.

## Data Availability

The raw data supporting the conclusions of this article will be made available by the authors, without undue reservation.
