# Supplementary material for: The relationship between uploader identity and the quality and reliability of depression-related Chinese short videos on TikTok and Bilibili
Source: Front Digit Health. 2026 Jul 27;8:1886505. doi: 10.3389/fdgth.2026.1886505 (PMC13454896; doi:10.3389/fdgth.2026.1886505)
Supplement: Supplementary file 1 [file Table1.docx]

Supplementary Material

**Supplementary Table A** Categorical data of video content on TikTok and Bilibili.

| **Variables** | **Bilibili (n =75)** | **TikTok (n =75)** |
| --- | --- | --- |
| **Video content(n) (%)** |  |  |
| Expert Psychoeducation | 24(32.00%) | 18(24.00%) |
| Lived Experience Narratives | 20(26.67%) | 22(29.33%) |
| Social Advocacy | 8(10.67%) | 11(14.67%) |
| High-Functioning Depression | 3(4.00%) | 5(6.67%) |
| Treatment Modalities | 7(9.33%) | 11(14.67%) |
| Metaphorical Representations | 10(13.33%) | 6(8.00%) |
| Non-Credible Content | 3(4.00%) | 2(2.67%) |
